# An Exploratory In Silico Analysis of *Chlamydia trachomatis*-Induced Inflammatory, Interferon, and ECM Transcriptional Programs and Their Translational Context in TCGA Ovarian Cancer

**DOI:** 10.3390/cancers18121920

**Published:** 2026-06-12

**Authors:** Rafaela Rodrigues, Carlos Sousa, Nuno Vale

**Affiliations:** 1 PerMed Research Group, RISE-Health, Faculty of Medicine, University of Porto, Alameda Professor Hernâni Monteiro, 4200-319 Porto, Portugal; up201908616@edu.med.up.pt (R.R.); carlos.sousa@synlab.pt (C.S.); 2 Molecular Biology Department, SYNLAB Portugal, Rua Manuel Pinto de Azevedo, 401, 4100-321 Porto, Portugal; 3 RISE-Health, Department of Community Medicine, Health Information and Decision (MEDCIDS), Faculty of Medicine, University of Porto, Rua Doutor Plácido da Costa, 4200-450 Porto, Portugal; 4 Laboratory of Personalized Medicine, Department of Community Medicine, Health Information and Decision (MEDCIDS), Faculty of Medicine, University of Porto, Rua Doutor Plácido da Costa, 4200-450 Porto, Portugal

**Keywords:** female tumorigenesis, fallopian tube, transcriptomics, TCGA, bioinformatics, *Chlamydia trachomatis*

## Abstract

**Background/Objectives**: *Chlamydia trachomatis* (CT) is a prevalent sexually transmitted pathogen associated with pelvic inflammatory disease, infertility, and has been proposed as a potential contributor to carcinogenesis through chronic inflammation and tissue remodeling. The molecular mechanisms triggered by CT infection in fallopian tube cellular contexts and their relevance to ovarian cancer transcriptomes remain incompletely understood. **Methods**: We analyzed GSE109428, profiling primary human fallopian tube mesenchymal cells infected with CT, to identify differentially expressed genes and characterize affected pathways using g:Profiler and STRING protein–protein association networks (confidence ≥ 0.7). To provide translational context, we computed ssGSEA scores in TCGA-OV for four signatures capturing IFN/ISG, TNF/NF-κB, NOD/innate immunity, and ECM programs, and evaluated inter-signature correlations and exploratory associations with overall survival (OS) and progression-free interval (PFI). **Results**: CT infection induced sustained inflammatory and interferon-associated transcriptional programs, with STRING networks highlighting cytokine hubs and a densely connected ISG module. Genes downregulated at 48 h post-infection (48-hpi) showed coherent enrichment for ECM organization and adhesion pathways. In TCGA-OV (*n* = 307), inflammatory and innate immune signatures co-occurred across tumors, with moderate correlations between TNF/NF-κB and NOD/innate (ρ = 0.591) and IFN/ISG and NOD/innate (ρ = 0.534). Exploratory survival analyses showed no significant associations with OS or PFI in Kaplan–Meier analyses or multivariable Cox models, including clinically adjusted and tumor microenvironment-adjusted specifications. **Conclusions**: CT infection induces sustained inflammatory and interferon-linked programs and coordinated repression of ECM networks in fallopian tube mesenchymal cells. Analogous immune transcriptional states co-occur in ovarian tumors, though the signatures evaluated did not yield robust prognostic signals in TCGA-OV. As this is an entirely in silico study without experimental validation, these findings should be treated as hypothesis-generating; thus, further mechanistic and experimental studies are warranted to clarify how CT infection-associated pathways may intersect with female tumorigenesis.

## Simple Summary

*Chlamydia trachomatis* (CT) is a common sexually transmitted infection that has been proposed as a potential contributor to female reproductive tract tumors through mechanisms such as chronic inflammation and tissue remodeling. To explore this hypothesis, we analyzed publicly available gene expression data from fallopian tube mesenchymal cells infected with CT and characterized the transcriptional signaling pathways modulated by infection, including inflammatory, interferon, and extracellular matrix pathways. To provide translational context, we examined whether analogous gene expression patterns are detectable in ovarian tumors from The Cancer Genome Atlas (TCGA). Our results show that CT infection induces sustained inflammatory and interferon responses and suppresses extracellular matrix programs in these cells, and that similar immune transcriptional states co-occur in ovarian tumors. These findings are hypothesis-generating and support further experimental investigation into the potential role of CT infection in ovarian cancer biology, though no causal relationship can be inferred from these data alone. Experimental validation of the identified candidate pathways will be an important next step.

## 1. Introduction

Inflammation is a well-recognized hallmark of cancer and is implicated across multiple stages of tumor development [1,2]. This immune-response signaling can be triggered by a variety of stimuli, arising from exogenous factors, particularly by infectious agents or endogenous sources including tissue damage, cellular stress, and dysregulated cellular signaling [3,4]. When inflammation becomes persistent, it may contribute to tumorigenesis by sustaining cytokine and chemokine signaling, increasing oxidative and replicative stress, and inducing microenvironmental alterations, such as stromal activation and extracellular matrix (ECM) remodeling [5,6,7,8]. Altogether, these processes may contribute to a pro-tumorigenic milieu that has been associated with tumor initiation and progression [9,10].

Within the female reproductive tract, several pathogens can drive inflammatory responses; among them, the bacterium *Chlamydia trachomatis* (CT) is one of the most common sexually transmitted infections worldwide [11,12,13,14,15]. CT can establish persistent or recurrent infections and is strongly associated with adverse reproductive outcomes, including pelvic inflammatory disease (PID), tubal scarring, and infertility [14,16,17]. Beyond reproductive morbidity, CT has been proposed as a potential contributor to pro-tumoral microenvironment development, through sustained inflammatory signaling, activation of innate immune pathways, metabolic and oxidative stress, and tissue remodeling [18,19,20,21,22,23]. However, the infection-induced molecular pathways that could plausibly connect CT exposure to tumor initiation and progression remain incompletely investigated [24].

The fallopian tube is a relevant reproductive tissue for investigating CT infection-associated mechanisms in gynecologic disease [25,26,27]. Indeed, mesenchymal cells in the fallopian tube contribute to ECM deposition, wound healing responses, and stromal remodeling, and they participate in the inflammatory crosstalk during tissue injury and repair [26]. In the setting of CT infection, stromal and mesenchymal responses may therefore contribute to chronic inflammation and fibrosis that underlie tubal pathology [28]. Characterizing transcriptional responses in fallopian tube mesenchymal cells can provide mechanistic insight into host pathways engaged by CT infection and into tissue remodeling pathways that may have downstream relevance to disease [29].

Interestingly, high-grade serous ovarian carcinoma (HGSOC), the most frequent and lethal ovarian cancer subtype, is strongly associated with the distal fallopian tube as a site of origin in many patients [30]. This anatomical and biological connection motivates investigating whether CT infection-associated transcriptional programs observed in fallopian tube cell types show overlap with transcriptional states present in ovarian tumors [30,31,32,33,34,35]. Importantly, pathway-level overlaps do not indicate causality or confirm pathogen presence in tumor tissues, but may provide translational context for mechanistic hypotheses.

Despite already established interest in this topic, evidence connecting CT infection to ovarian tumorigenesis remains limited and has not been systematically explored at the level of infection-induced transcriptional programs and their relevance in the tumoral context [36,37]. In this study, we performed an integrative in silico analysis using publicly available transcriptomic data from primary human fallopian tube mesenchymal cells infected in vitro with CT (GSE109428) to identify differentially expressed genes and to characterize affected biological processes using functional enrichment and protein-protein association network analysis [38]. To provide transcriptional context, we further analyzed TCGA ovarian cancer (TCGA-OV) by computing single-sample gene set enrichment (ssGSEA) scores for four pre-defined signatures capturing IFN/ISG, TNF/NF-kB, NOD/innate immunity, and ECM programs, and by evaluating inter-signature relationships and exploratory associations with clinical outcomes [39]. This study design is based on the re-analysis of existing high-throughput datasets to generate testable hypotheses and prioritize candidate pathways for future mechanistic validation using approaches that can distinguish contributions from different cell populations [40,41,42]. Accordingly, we aimed to define CT-responsive gene expression changes in primary human fallopian tube mesenchymal cells and identify enriched biological processes and interaction modules using g:Profiler and STRING [43,44]. Concomitantly, we assessed the co-occurrence and exploratory prognostic relevance of selected infection-linked signatures in TCGA ovarian tumors (TCGA-OV) using ssGSEA and survival analyses [45].

**Figure 1:**
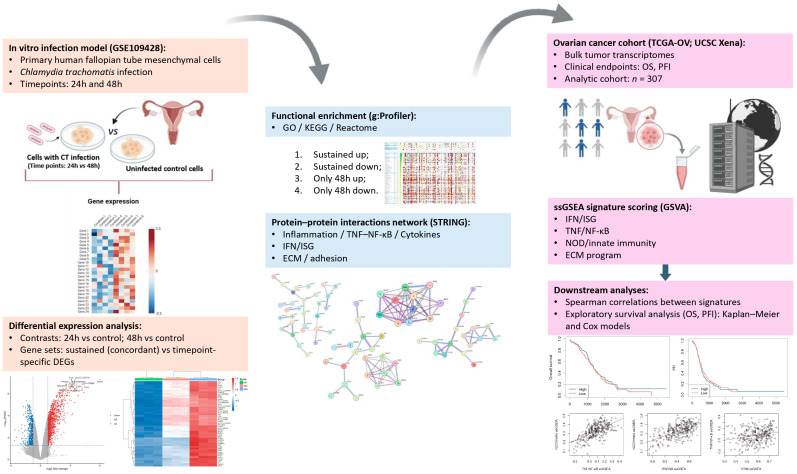
Graphical abstract—overview of the study design and analysis workflow. Transcriptomic data from CT-infected primary human fallopian tube mesenchymal cells (GSE109428; 24 h and 48 h) were analyzed to identify differentially expressed genes and derive sustained and timepoint-specific gene sets. Functional interpretation was performed using g:Profiler enrichment and STRING protein–protein association networks. To provide translational context, ssGSEA (GSVA) signature scores were computed in TCGA ovarian cancer (TCGA-OV; UCSC Xena; analytic cohort, *n* = 307) and evaluated using inter-signature correlations and exploratory survival analyses (OS and PFI). Abbreviations: CT, *Chlamydia trachomatis*; DEGs, differentially expressed genes; TNF, tumor necrosis factor; NF-kB, nuclear factor kappa B; IFN, interferon; ISG, interferon-stimulated genes; ECM, extracellular matrix; OS, overall survival; PFI, progression-free interval. Definitions: sustained, significant at both timepoints with concordant direction. Figure created in BioRender. Rafaela Rodrigues (2026) https://www.biorender.com/.

A schematic overview of the study design is provided in Figure 1, summarizing the in vitro infection transcriptome analysis, functional/network interpretation, and the translational ssGSEA-based evaluation in TCGA-OV.

## 2. Materials and Methods

### 2.1. Transcriptomic Data Search Strategy

The public transcriptomic datasets related to CT infection were searched in the NCBI Gene Expression Omnibus (GEO) database [46]. The query term “*Chlamydia trachomatis*” was used and results were filtered to “*Homo sapiens*” origin and study type “Expression profiling by array”. This search returned 28 datasets, which were then screened manually based on predefined inclusion/exclusion criteria aligned with the study objective of characterizing host transcriptional programs induced by CT infection in a fallopian tube–relevant cellular context. In detail, the inclusion criteria were human-derived samples; a clear CT infection vs. an appropriate uninfected control; availability of raw or processed expression data with sample-level metadata sufficient to define groups; and a study design compatible with differential expression analysis without major co-interventions. Additionally, the exclusion criteria were blood samples; studies involving drug treatments or additional interventions beyond CT infection; established long-term cell lines if primary tissue-derived cells were available; and non-gynecologic infection contexts.

After the manual screening, GSE109428 dataset (“Primary mesenchymal cells from the human fallopian tube infected with *Chlamydia trachomatis*”) was selected because it uses primary human fallopian tube mesenchymal cells, which are a stromal population relevant to tissue remodeling and scarring, also it includes defined timepoints: 24- and 48- hours post-infection (hpi) enabling evaluation of initial and sustained responses, and finally, it provides a direct infection vs. control workflow, appropriate for downstream analyses [38]. Importantly, this dataset has been comparatively underexplored in the context of CT infection and tumorigenesis hypothesis generation.

### 2.2. Differential Gene Expression Analysis

Differential gene expression analysis was performed in R version 4.5.3 (2026-03-11 ucrt) using the Bioconductor package Linear Models for Microarray Data (limma) (version 3.66.0). The GSE109428 series matrix was retrieved using the GEOquery package (version 2.78.0). Prior to differential expression analysis, quality control was performed to assess data integrity and sample consistency. Expression values distributions were inspected across samples. Expression values were log2-transformed when required (maximum expression value > 100). Unsupervised principal component analysis (PCA) was performed on the full expression matrix to assess sample clustering by experimental group and to identify potential outliers or batch effects. All nine samples clustered in accordance with their assigned experimental groups (control, 24-hpi, 48-hpi), with no evidence of outliers or systematic batch effects. Probes with zero variance across samples were removed prior to model fitting. It should be noted that the GSE109428 dataset includes a single set of non-infected control samples (*n* = 3) used as the reference for both the 24-hpi and 48-hpi comparisons, as time-matched controls were not available in this public dataset. Consequently, transcriptional changes observed at each timepoint are interpreted relative to the same baseline, and the potential contribution of culture duration effects cannot be formally excluded.

A design matrix was defined to model the three experimental conditions (uninfected control, 24- and 48-hpi). Linear models were fitted using lmFit (limma package version 3.66.0), and contrasts were specified to compare infected samples at 24- and 48-hpi against controls. Empirical Bayes moderation was applied using eBayes (limma package version 3.66.0). Multiple testing correction was performed using the Benjamini–Hochberg false discovery rate (FDR) method.

Microarray probe identifiers were mapped to gene symbols using the corresponding GEO platform annotation file (GPL21272, Agilent microarray, Santa Clara, CA, USA) [47]. Probes without valid gene symbol annotations were excluded. When multiple probes mapped to the same gene symbol, probe-level results were collapsed to gene level by retaining the probe with the lowest adjusted *p*-value for that gene. Differentially expressed genes (DEGs) were defined using an adjusted *p*-value < 0.05 and an absolute log2 fold change (|log2FC|) ≥ 1. To facilitate interpretation and address reviewer requests for transparency, the top 25 upregulated and downregulated genes at each timepoint, ranked by FDR, are provided in Supplementary File S1 (Table S1), including log2FC, mean expression, t-statistic, and FDR values. DEG sets were subsequently used for functional enrichment and protein–protein association network analyses. All data processing, statistical analysis, integration, and visualization steps were performed in R. The complete R script is provided in Supplementary File S1.

### 2.3. Functional Enrichment Analysis

Functional enrichment analysis was performed to interpret the biological processes represented in the CT-responsive gene sets derived from the previous differential expression analysis. In detail, this analysis was carried out using the g:Profiler web server (g:GOSt), with the organism set to *Homo sapiens* [44]. To reduce platform-related bias, the enrichment background or gene universe was restricted to genes represented on the GPL21272 microarray platform, mapped to valid gene symbols after preprocessing and annotation (*n* = 32,063). Gene Ontology (GO) Biological Processes (GO:BP), KEGG, and Reactome terms were queried. Also, multiple testing correction using the g:SCS (Set Counts and Sizes) method implemented in g:Profiler, which controls for multiple testing in the context of gene set enrichment and is not equivalent to Benjamini–Hochberg FDR correction. Significantly enriched terms were defined at a g:SCS-adjusted *p*-value threshold of <0.05. For reporting in the main text, a more stringent threshold of g:SCS-adjusted *p*-value < 0.01 was applied to prioritize the most robust terms. Enrichment results were used to guide the selection of representative gene signatures for downstream analyses and to support interpretation of infection-associated transcriptional programs.

### 2.4. Protein–Protein Interaction (PPI) Analysis

PPI analysis was performed using the Search Tool for the Retrieval of Interacting Genes/Proteins (STRING) web platform (version 12.0) to identify interaction modules and highly connected pathways within CT-responsive gene sets [43]. Networks were generated using a high-confidence interaction threshold (combined score ≥ 0.7), restricted to *Homo sapiens*. To minimize literature-driven and context-independent links, text-mining and genomic neighborhood evidence were excluded. The resulting PPI networks were visualized to assess dominant biological pathways, interaction patterns, and functional modules.

### 2.5. TCGA-OV ssGSEA Signature Scoring, Tumor Microenvironment Deconvolution, and Survival Analysis

As a translational extension of the in vitro CT infection analysis, we investigated whether infection-linked transcriptional programs are reflected as expression signatures in human ovarian tumors and whether they show any exploratory association with clinical outcomes. Gene-level mRNA expression data for ovarian cancer were retrieved from the UCSC Xena platform using the UCSCXenaTools R package (version 1.7.0). We analyzed the TCGA-OV HiSeqV2 gene expression matrix (UCSC Xena dataset identifier: TCGA.OV.sampleMap/HiSeqV2). In detail, TCGA-OV was selected given the relevance of the fallopian tube to high-grade serous ovarian carcinoma and to provide a clinically annotated tumor transcriptome resource for signature-based analyses.

The downloaded expression matrix was processed as a gene-by-sample matrix with gene symbols as row names and log2-scaled expression values as provided by UCSC Xena. Four biologically motivated gene signatures were defined based on infection-associated programs observed in the in vitro analysis. Signature gene selection followed a data-driven approach based on the results of the in vitro transcriptomic analysis. Briefly, following differential expression analysis, each DEG set was independently submitted to g:Profiler functional enrichment analysis. Enriched biological terms were reviewed, and non-redundant representative pathways were selected across GO:BP, KEGG, and Reactome databases. For each selected pathway, the corresponding contributing genes from the DEG list were identified, and subsets of these genes were submitted to STRING network analysis to identify highly connected interaction modules. Signature genes were then defined as those forming coherent, densely connected modules in STRING that were also supported by functional enrichment evidence, ensuring that each signature reflected a biologically interpretable and network-supported transcriptional program rather than an arbitrary gene list. Specifically, the IFN/ISG signature (12 genes) was derived from the interferon/ISG STRING module of sustained upregulated genes, comprising *MX1*, *MX2*, *IFIT1*, *IFIT2*, *IFIT3*, *OAS2*, *OASL*, *ISG15*, *USP18*, *RSAD2*, *IRF7*, and *ISG20*. The TNF/NF-κB signature (13 genes) was derived from the cytokine/chemokine STRING module, comprising *IL1A*, *IL1B*, *IL6*, *CXCL1*, *CXCL2*, *CCL2*, *CCL3*, *CCL5*, *NFKBIA*, *RELB*, *NFKB1*, *TNF*, and *PTGS2*. The NOD/innate signature (9 genes) was derived from innate immune signaling components enriched in the sustained upregulated set, comprising *RIPK2*, *TRAF6*, *NFKB1*, *IRF9*, *CASP4*, *GBP5*, *NLRP3*, *SQSTM1*, and *PELI2*. The ECM signature (15 genes) was derived from the basement membrane and the ECM STRING module of genes downregulated exclusively at 48-hpi, comprising *COL1A2*, *COL3A1*, *COL4A1*, *COL4A2*, *COL5A1*, *COL5A2*, *LAMA2*, *LAMB1*, *LAMB2*, *LAMC1*, *NID1*, *NID2*, *POSTN*, *FN1*, and *ITGAV*. Prior to scoring, each signature was intersected with the TCGA-OV expression matrix to retain only genes present in the dataset, and the final signature sizes were recorded. For each tumor sample, signature scores were calculated using single-sample gene set enrichment analysis (ssGSEA) implemented in the GSVA package (parameter-based API; ssgseaParam), generating one score per signature, per tumor sample. Associations between signature scores were assessed using Spearman’s rank correlation with pairwise complete observations.

To characterize the contribution of tumor microenvironment composition to the observed signature scores, immune and stromal infiltration were estimated using the ESTIMATE algorithm, implemented in the ESTIMATE R package. Briefly, ESTIMATE derives an ImmuneScore and a StromalScore for each sample based on the expression of curated immune- and stromal-associated gene sets, respectively, alongside a composite ESTIMATEScore. The expression matrix was formatted according to ESTIMATE requirements and filtered to the 10,412 common genes using filterCommonGenes prior to score computation. Spearman correlations between ssGSEA signature scores and ESTIMATE scores were computed and visualized to assess the extent to which signature variation reflects immune or stromal cell abundance.

Clinical endpoints were obtained from the UCSC Xena curated survival table (dataset identifier: survival/OV_survival.txt), including overall survival (OS) and progression-free interval (PFI). Clinical covariate data, including age at diagnosis, tumor stage (FIGO), histological grade, and residual disease, were retrieved from the TCGA-OV clinical matrix (UCSC Xena dataset identifier: TCGA.OV.sampleMap/OV_clinicalMatrix). Stage was categorized as I–II, III, or IV; grade as G1–G2 or G3–G4; and residual disease as none, low (1–10 mm), or high (>10 mm).

For exploratory visualization, tumors were stratified into “High” versus “Low” groups based on a median split of a signature score (IFN/ISG), and Kaplan–Meier curves were compared using the log-rank test. Multivariable Cox proportional hazards models were fitted for OS and PFI in two specifications: (i) an unadjusted model including only the four continuous ssGSEA signature scores, and (ii) a clinically adjusted model additionally including age, stage, grade, and residual disease as covariates. A third model further incorporated ESTIMATE ImmuneScore and StromalScore as additional covariates to account for tumor microenvironment composition. The analytic cohort for adjusted models was restricted to samples with complete data for all covariates (*n* = 264; 43 samples excluded due to missing clinical covariate data, primarily residual disease status). To account for multiple comparisons across signatures and endpoints, *p*-values from all Cox models were adjusted using the Benjamini–Hochberg (BH) false discovery rate procedure. All analyses were performed in R and for reproducibility, the full workflow is provided in Supplementary File S2.

### 2.6. Statistical Analysis and Visualization

All analyses were performed in RStudio (version 2025.09.1+401). Differential expression analyses were conducted using limma with empirical Bayes moderation and Benjamini–Hochberg false discovery rate (FDR) correction. For the microarray dataset, expression values were log2-transformed when required (maximum expression value > 100), and probes with zero variance across samples were removed prior to model fitting. Probe identifiers were mapped to gene symbols using the GEO platform annotation (GPL21272) and probes without valid gene symbols were excluded. When multiple probes were mapped to the same gene symbol, results were collapsed to the gene level by retaining the probe with the lowest adjusted *p*-value for that gene. DEGs were defined using FDR < 0.05 and |log2FC| ≥ 1. Functional enrichment analysis was performed using g:Profiler with g:SCS-adjusted *p*-values. STRING network analysis used a high-confidence threshold (combined score ≥ 0.7), excluding text-mining and genomic neighborhood evidence.

Data manipulation was performed using the dplyr package. Volcano plots were generated using ggplot2 and heatmaps using pheatmap. TCGA-OV data retrieval was performed using UCSCXenaTools; ssGSEA scoring used the GSVA package; correlation analyses used Spearman’s method; tumor microenvironment deconvolution was performed using the ESTIMATE package, deriving ImmuneScore, StromalScore, and ESTIMATEScore for each sample; survival analyses used the survival and survminer packages, including Kaplan–Meier curves with log-rank tests and Cox proportional hazards models. Multivariable Cox models were fitted in two specifications: unadjusted (signature scores only) and clinically adjusted (additionally incorporating age, tumor stage, histological grade, and residual disease), with a third specification further including ESTIMATE ImmuneScore and StromalScore. To account for multiple comparisons across signatures and endpoints, *p*-values from Cox models were adjusted using the Benjamini–Hochberg procedure.

All scripts used to reproduce these analyses and generate figures are provided in Supplementary Files: S1—differential expression pipeline; Supplementary Files: S2—TCGA-OV ssGSEA, ESTIMATE, and survival pipeline.

## 3. Results

### 3.1. Differential Expression Genes Following CT Infection

Differential expression analysis of the GSE109428 dataset, entitled “Primary mesenchymal cells from human fallopian tube infected with *Chlamydia trachomatis*”, from da Costa AT, Mollenkopf H, Meyer TF, and Berger H (Table 1), identified broad transcriptional alterations following CT infection at both timepoints [38].

Using an FDR < 0.05 and |log2FC| ≥ 1 threshold, we detected 238 upregulated and 10 downregulated genes at 24-hpi relative to uninfected controls, and 1112 upregulated and 463 downregulated genes at 48-hpi. To distinguish persistent from timepoint-specific changes, we defined “sustained” genes as those significantly differentially expressed at both timepoints with concordant direction, yielding 223 sustained upregulated genes and 6 sustained downregulated genes. Genes significant only at 48-hpi (and not at 24-hpi under the same criteria) were considered a later, timepoint-specific transcriptional response and comprised 889 upregulated and 457 downregulated genes. We prioritized sustained and 48-hpi-only gene sets for downstream enrichment and network analyses because they represent transcriptional programs that persist or emerge after prolonged infection in this experimental system. It should be noted, however, that these timepoints represent early in vitro responses to CT infection and do not directly model long-term or chronic infection dynamics. The potential relevance of these programs to long-term sequelae such as chronic inflammation, fibrosis, or tumorigenesis is speculative and is proposed as a hypothesis for future investigation rather than a conclusion supported by the current data. A summary of gene set sizes is shown in Table 2, and complete gene lists are provided in Supplementary File S1.

To facilitate interpretation and transparency, the top 25 upregulated and downregulated DEGs at each timepoint, ranked by FDR, are additionally provided in Supplementary File S1 (Table S1), including log2FC, mean expression, t-statistic, and FDR values. Among the most significantly upregulated genes at 24-hpi were *EGR4*, *ATF3*, *IL1A*, *RSAD2*, and *OASL*, while at 48-hpi the top upregulated genes included *EGR4*, *PTGS2*, *IL1A*, *FOS*, *CSF2*, and *OASL*, consistent with progressive amplification of inflammatory and interferon-associated molecular pathways. The most significantly downregulated genes at 48-hpi included *HSPG2*, *COL12A1*, *TNXB*, *ITGA8*, and *ITGAV*, consistent with coordinated repression of ECM and adhesion programs.

Subsequently, each DEG gene set (sustained up, 48-hpi-only up, and 48-hpi-only down) was analyzed using g:Profiler to identify enriched biological terms. In detail, to summarize enrichment results of g:Profiler outputs, we prioritized the most robust terms (g:SCS-adjusted *p*-value < 0.01) for reporting in the main text, which were also supported by a meaningful overlap between the query list and the annotated term (intersection size), while considering term size to avoid overly broad annotations. When multiple related terms were returned, we focused on non-redundant, biologically interpretable categories and used STRING to contextualize these themes at the level of connected gene modules. Given the small number of genes, the sustained downregulated set (*n* = 6) was not emphasized in enrichment summaries.

### 3.2. Sustained Upregulated Genes Cluster into Inflammatory and Interferon-Associated Network Modules

Functional enrichment of the sustained upregulated gene set identified inflammation- and cytokine-related biology (Table 3). Enriched GO:BP terms included cellular response to IL1 (GO:0071347) and negative regulation of MAPK cascade (GO:0043409), indicating over-representation of cytokine-responsive signaling and MAPK regulatory annotations. KEGG enrichment indicated over-representation of inflammatory signaling pathways, specifically, TNF signaling (KEGG:04668), IL17 signaling (KEGG:04657), cytokine–cytokine receptor interaction (KEGG:04060), MAPK signaling (KEGG:04010), and NF-κB signaling (KEGG:04064). These pathways were supported by multiple genes from the DEG list mapping to each pathway (8 to 16 genes per pathway among the terms reported). Reactome enrichment further supported interleukin signaling with IL10 signaling (R-HSA-6783783), IL4 and IL13 signaling (R-HSA-6785807), and a prominent type I interferon module, IFN-α/β signaling (R-HSA-909733).

**Figure 2:**
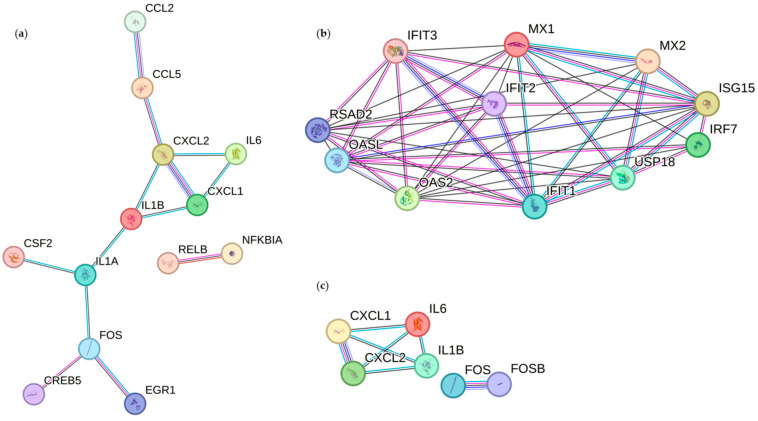
STRING protein–protein association networks of sustained upregulated genes following CT infection. Networks were generated using STRING (v12.0; *Homo sapiens*) with a high-confidence threshold (combined score ≥ 0.7) and excluding text-mining and genomic neighborhood evidence. Nodes represent proteins and edges represent known or predicted functional associations derived from multiple evidence channels, and do not exclusively represent experimentally validated protein–protein interactions. Panel (**a**) shows the TNF/NF-κB/cytokine–chemokine signaling module, centered on hub nodes IL1A and IL1B, with chemokine components CXCL1/CXCL2 and CCL2/CCL5, and NF-κB regulatory components RELB and NFKBIA. Panel (**b**) shows the interferon/ISG module, a densely connected network centered on hub nodes MX1 and ISG15, comprising IFIT1/2/3, OAS2/OASL, USP18, RSAD2, MX2, and IRF7. Panel (**c**) shows the IL-17-related inflammatory module, centered on IL6 and IL1B, with chemokine components CXCL1/CXCL2 and transcription factor components FOS/FOSB. Figure generated using STRING (https://string-db.org/; accessed on 28 May 2026).

Consistent with the enrichment results, STRING network analysis highlighted distinct connected modules within the sustained upregulated gene set (Figure 2). Panel A shows a cytokine/chemokine network centered on IL1A/IL1B, chemokines (CXCL1/CXCL2, CCL2/CCL5), and IL6, with an additional regulatory component of NF-κB signaling, involving NFKBIA and RELB. Panel B shows a highly connected interferon/ISG module comprising MX1/MX2, IFIT1/IFIT2/IFIT3, OAS2/OASL, ISG15, USP18, RSAD2, and IRF7. Panel C highlights an IL17-associated inflammatory module in which chemokines (CXCL1/CXCL2) connect to key cytokine nodes (IL1B, IL6). Additionally, FOS and FOSB form a small, isolated two-node submodule within this network.

Together, these enrichment and network results support sustained upregulation of inflammatory and interferon-linked gene expression signatures following CT infection.

### 3.3. Late Downregulated Response at 48-hpi Is Dominated by ECM and Adhesion Programs

Genes downregulated exclusively at 48-hpi showed strong and coherent enrichment for ECM and adhesion-related functions (Table 4). GO:BP terms were dominated by extracellular matrix organization (GO:0030198). KEGG pathways included ECM–receptor interaction (KEGG:04512) and focal adhesion (KEGG:04510), supporting coordinated changes in cell–matrix interaction programs. A cancer-labeled KEGG term, small cell lung cancer (KEGG:05222), was also enriched, likely reflecting shared core ECM/adhesion and signaling components rather than tissue-specific disease inference. Reactome further supported a motility/adhesion-associated signature through MET and PTK2 signaling (REAC:R-HSA-8874081 and REAC:R-HSA-6806834).

**Figure 3:**
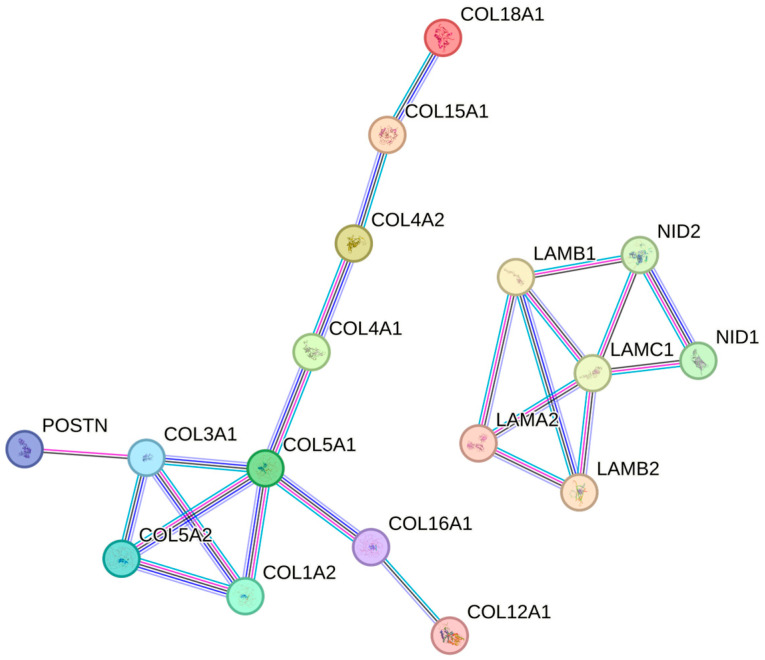
STRING protein–protein association networks of genes downregulated exclusively at 48-hpi. Networks were generated using STRING (v12.0; *Homo sapiens*) with a high-confidence threshold (combined score ≥ 0.7) and excluding text-mining and genomic neighborhood evidence. Nodes represent proteins and edges represent known or predicted functional associations derived from multiple evidence channels, and do not exclusively represent experimentally validated protein–protein interactions. The dominant module shows a densely connected ECM/basement membrane network centered on collagen family members (*COL4A1*, *COL4A2*, *COL1A2*, *COL3A1*, *COL5A1/2*), laminin subunits (*LAMA2*, *LAMB1/2*, *LAMC1*), and nidogens (*NID1*, *NID2*), consistent with coordinated repression of ECM structural and adhesion programs at 48-hpi. Figure generated using STRING (https://string-db.org/; accessed on 28 May 2026).

STRING network analysis (Figure 3) confirmed a densely connected ECM/basement membrane module dominated by collagens and basement membrane components, such as COL family members, laminins, and nidogens, consistent with coordinated repression of ECM structural and adhesion-related networks at 48-hpi.

### 3.4. Late Upregulated Response at 48-hpi Highlights Cytokine and Immune Signaling Pathways

Genes upregulated exclusively at 48-hpi were enriched for immune and cytokine-driven signaling (Table 5). KEGG terms included transcriptional misregulation in cancer (KEGG:05202), and cytokine–cytokine receptor interaction (KEGG:04060), while Reactome highlighted signaling by interleukins (REAC:R-HSA-449147) and IL4 and IL13 signaling (REAC:R-HSA-6785807).

**Figure 4:**
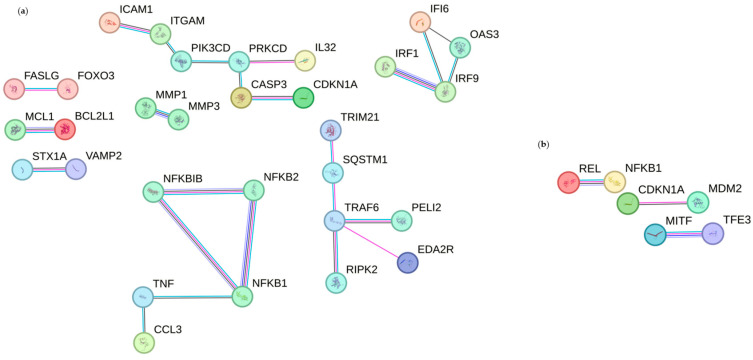
STRING protein–protein association networks of genes upregulated exclusively at 48-hpi (48-hpi-only up). Networks were generated using STRING (v12.0; *Homo sapiens*) with a high-confidence threshold (combined score ≥ 0.7) and excluding text-mining and genomic neighborhood evidence. Nodes represent proteins encoded by the input gene set and edges represent known or predicted functional associations derived from multiple evidence channels, and do not exclusively represent experimentally validated protein–protein interactions. Panel (**a**) shows a cytokine and innate immune signaling module centered on hub nodes NFKB1/2/IB and TNF, comprising TRAF6 and RIPK2, also CASP3 and related immune regulators. Panel (**b**) shows a cancer-associated transcriptional misregulation module centered on REL and NFKB1, also TFE3, MITF, and CDKN1A, MDM2. Figure generated using STRING (https://string-db.org/; accessed on 28 May 2026).

STRING network inspection supported connected immune signaling components, consistent with a late transcriptional program characterized by cytokine-mediated communication and innate immune activation (Figure 4).

### 3.5. TCGA-OV ssGSEA Suggests Co-Occurrence of Inflammatory and Innate Immune Programs and Signature Scores Show No Independent Prognostic Association After Clinical and Microenvironmental Adjustment

To evaluate whether infection-linked programs show analogous transcriptional states in ovarian tumors, we computed ssGSEA scores in TCGA-OV for four signatures representing IFN/ISG (12 genes), TNF/NF-κB (13 genes), NOD/innate immunity (9 genes), and ECM (15 genes) (analytic cohort *n* = 307).

**Figure 5:**
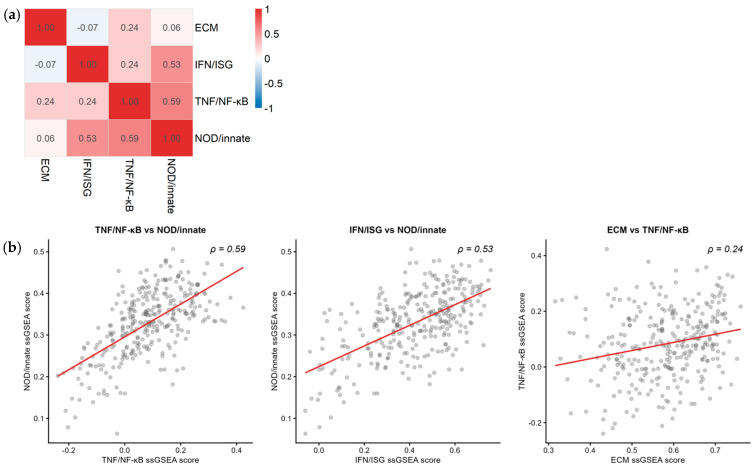
Inter-signature relationships in TCGA-OV based on ssGSEA scores. The upper panel (**a**), shows a heatmap of Spearman correlations between ssGSEA signature scores for ECM, IFN/ISG, TNF/NF-κB, and NOD/innate programs across the TCGA-OV analytic cohort (*n* = 307). The lower panels (**b**), show representative pairwise scatterplots illustrating the moderate co-occurrence of inflammatory and innate immune programs (TNF/NF-κB vs. NOD/innate, ρ = 0.59; IFN/ISG vs. NOD/innate, ρ = 0.53) and the weak correlation between the ECM signature and inflammatory programs (ECM vs. TNF/NF-κB, ρ = 0.24), consistent with ECM variation being driven by distinct biological processes such as stromal composition rather than inflammatory signaling. Red lines indicate a fitted trend line for visualization.

Across tumors, inflammatory and innate immune programs co-varied: TNF/NF-κB and NOD/innate scores were moderately correlated (Spearman ρ = 0.59), and IFN/ISG also correlated with NOD/innate (ρ = 0.53). In contrast, the ECM signature showed weak-to-negligible correlations with IFN/ISG (ρ = −0.07) and NOD/innate (ρ = 0.06), suggesting partial independence of ECM variation from the inflammatory axes captured by these signatures (Figure 5).

To contextualize these patterns in terms of tumor microenvironment composition, we applied the ESTIMATE algorithm to derive ImmuneScore and StromalScore for each sample. The ECM signature showed a strong positive correlation with StromalScore (ρ = 0.83), indicating that ECM score variation in bulk tumor data is largely driven by stromal cell abundance rather than reflecting a specific infection-associated program. The TNF/NF-κB and NOD/innate signatures correlated substantially with ImmuneScore (ρ = 0.72 and 0.74, respectively), consistent with these scores partially capturing immune cell infiltration. The IFN/ISG signature showed weaker correlations with both StromalScore (ρ = 0.07) and ImmuneScore (ρ = 0.34), suggesting a relatively more specific transcriptional component.

**Figure 6:**
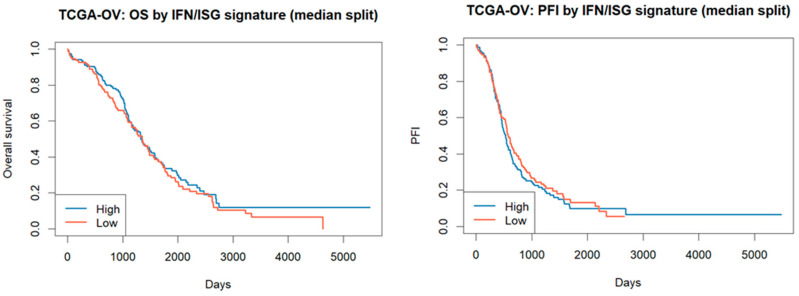
Exploratory survival analyses in TCGA-OV using the IFN/ISG ssGSEA signature score. Kaplan–Meier curves for overall survival (OS; **left panel**) and progression-free interval (PFI; **right panel**) are shown after stratifying tumors into IFN/ISG “high” and “low” groups using a median split of the IFN/ISG ssGSEA score (analytic cohort *n* = 307; OS events = 185; PFI events = 214). *p*-values were computed using the log-rank test.

In exploratory survival analyses, median stratification of the IFN/ISG score did not yield significant differences in OS (log-rank *p* = 0.4) or PFI (*p* = 0.5) (Figure 6). In the unadjusted multivariable Cox model including all four signature scores, no statistically significant independent associations with OS or PFI were observed, and the overall model tests were not significant (OS: likelihood ratio *p* = 0.6; PFI: *p* = 0.9).

In the clinically adjusted model (*n* = 264, after exclusion of samples with missing clinical covariate data; OS events = 160; PFI events = 186), including age, tumor stage, histological grade, and residual disease as covariates, none of the four signatures showed statistically significant independent associations with OS or PFI after BH correction (all FDR > 0.05). Age at diagnosis was the only variable with a nominally significant association with OS (HR = 1.03, 95% CI 1.01–1.04, *p* = 0.003; FDR = 0.057). Trends in the expected direction were observed for grade (HR = 1.61 for OS) and residual disease (HR = 1.57 for OS, low vs. none), consistent with their established prognostic roles, though these did not reach statistical significance in this cohort. When ESTIMATE ImmuneScore and StromalScore were further incorporated as covariates, results were unchanged and none of the signatures attained significance, confirming that the absence of prognostic signal is not attributable to confounding by tumor microenvironment composition (Table 6).

Multivariable Cox models including all four continuous signature scores showed no statistically significant independent associations with OS or PFI, and the overall model tests were not significant (OS: likelihood ratio *p* = 0.6; PFI: likelihood ratio *p* = 0.9), indicating that these short signature scores did not provide a robust prognostic signal in this setting (Table 6).

## 4. Discussion

In this exploratory in silico study, we characterized gene expression signatures induced by CT infection in primary human fallopian tube mesenchymal cells and evaluated whether analogous inflammatory and ECM-related programs are detectable in clinically annotated ovarian tumors from TCGA-OV [38,45,48]. By integrating differential expression analysis, functional enrichment, protein–protein interaction network analysis, and ssGSEA scoring, this work provides a reproducible, hypothesis-generating framework and prioritizes infection-associated pathways for follow-up in studies addressing potential links between CT infection, chronic tissue remodeling, and female tumorigenesis.

The most prominent transcriptional feature of CT-infected fallopian tube mesenchymal cells was a pronounced and sustained inflammatory response, characterized by enrichment of TNF/NF-κB, IL-17, and cytokine-mediated signaling pathways alongside a densely connected STRING module centered on IL1A/IL1B, IL6, CXCL1/2, CCL2/5, and related chemokines (Table 3 and Figure 2). These findings are consistent with the known immunobiology of CT infection, which triggers pattern recognition receptor activation and downstream cytokine cascades, and with broader enrichment of NF-κB- and MAPK-related terms together with cytokine–cytokine receptor interaction annotations [13]. The sustained nature of this response, detectable across both post-infection timepoints, is consistent with CT engaging host transcriptional programs characteristic of chronic inflammatory tissue states rather than a purely transient innate response.

In parallel, we observed a strongly connected interferon-stimulated gene (ISG) module, with enrichment for type I interferon signaling and a compact STRING subnetwork comprising MX1/MX2, IFIT1/2/3, OAS2/OASL, ISG15, USP18, RSAD2, and IRF7 (Figure 2). This pattern is consistent with activation downstream of intracellular pathogen detection and can persist beyond the early-infection timepoint. Persistent interferon-associated programs may have complex downstream consequences, including modulation of cytokine networks, antigen presentation, cell survival and stress responses [49,50,51]. Importantly, both the inflammatory and ISG programs have been implicated in chronic inflammation-associated carcinogenesis, where persistent cytokine signaling and oxidative stress can promote genomic instability and pro-tumorigenic microenvironmental changes [8,52,53]. In the context of infection–tumorigenesis hypotheses, these sustained inflammatory and interferon-linked axes represent a mechanistically plausible route by which recurrent or persistent CT infection could potentially contribute to long-term microenvironmental perturbation, even if active infection is no longer present at the time of tumor development. Indeed, the concurrent activation of interferon and NF-κB programs observed here is mechanistically supported by evidence from Madaan and colleagues, who demonstrate in fallopian tube epithelial cells that ISGylation, mediated by ISG15 and its activating enzyme UBA7, simultaneously amplifies IRF3 and NF-κB signaling downstream of cytosolic RIG-I/MDA5 pattern recognition receptors [54]. Although this mechanism was defined in epithelial cells, it suggests that coordinated interferon and NF-κB activation may represent a conserved feature of innate immune signaling across fallopian tube cellular compartments. In the context of intracellular infections, such as with CT, this suggests a plausible molecular basis for the co-regulation of these transcriptional axes observed in our data, consistent with transcriptional states that have been associated with pro-tumorigenic microenvironmental conditions in other contexts.

It should also be acknowledged that the relationship between chronic infection, inflammation, and tumorigenesis is not unidirectional. Persistent CT infection and the associated inflammatory and interferon responses could also promote immune surveillance mechanisms or induce fibrotic responses that may inhibit early neoplastic transformation. The net effect of CT-associated stromal and immune perturbation on tumor initiation or progression is therefore uncertain, and it depends on the context, which underscores the need for experimental models that can clarify these biological processes.

A second major observation of our analysis was the coherent downregulation of ECM and adhesion-related programs specifically at 48-hpi, including enrichment for ECM organization, ECM–receptor interaction, and focal adhesion pathways, and a densely connected basement membrane STRING module dominated by collagens, laminins, and nidogens (Table 4 and Figure 3). Reactome enrichment for MET–PTK2 signaling further supports involvement of motility- and adhesion-associated signaling axes [55,56]. Mesenchymal and stromal cells are central orchestrators of tissue remodeling and fibrosis; thus, coordinated downregulation of ECM structural genes in this cell type suggests that CT infection may perturb stromal programs that regulate tissue architecture and cell–matrix interactions [57]. This finding aligns with reports that CT infection induces stromal activation and fibrotic responses in the fallopian tube, contributing to tubal scarring and dysfunction [27,58,59]. In cancer biology, ECM remodeling and altered adhesion signaling are key features of tumor microenvironments that influence invasion, immune cell trafficking, and therapeutic resistance [60,61,62,63].

The downregulation of ECM structural genes in CT-infected mesenchymal cells at 48-hpi may appear paradoxical given that most solid tumors, including HGSOC, are associated with ECM upregulation and desmoplasia [64]. This apparent contrast may reflect several non-mutually exclusive possibilities: an acute cytoskeletal reorganization response to intracellular infection, a transient adaptation that precedes subsequent fibrotic remodeling upon resolution or persistence of infection, or a limitation of the in vitro system that cannot recapitulate the complexity of a multicellular in vivo system [65,66]. Importantly, fibrotic remodeling in the context of CT infection is a complex process involving immune cell recruitment and paracrine cytokine signaling that cannot be fully modeled in a single-cell infection system. Whether the acute ECM repression observed here is protective, permissive, or a transient in vitro response warrants investigation in more physiologically relevant experimental models.

To provide translational tumor context, we quantified ssGSEA scores for infection-linked inflammatory, innate, interferon, and ECM programs in TCGA-OV. The moderate correlations observed between TNF/NF-κB, NOD/innate, and IFN/ISG scores indicate that these immune-related transcriptional states frequently co-occur across ovarian tumor bulk transcriptomes, consistent with the concept of an inflamed tumor microenvironment phenotype described across multiple cancer types and associated with immune cell infiltration and coordinated cytokine signaling [67,68,69]. This pattern may reflect combined contributions from tumor cells and the immune and stromal components of the tumor microenvironment [67]. Notably, the ECM signature showed weak correlation with inflammatory programs, suggesting that ECM variation in tumors is likely driven by distinct biological processes, such as stromal composition, desmoplastic responses, or fibrosis, rather than by the same inflammatory axes captured here, consistent with the known functional heterogeneity of cancer-associated fibroblasts and their role in extracellular matrix remodeling and desmoplasia [64,70,71,72]. It should be emphasized that the co-occurrence of inflammatory transcriptional states in TCGA-OV tumors reflects broad immune programs that are common across many cancer types and inflammatory conditions and cannot be interpreted as evidence of CT-driven oncogenesis or pathogen presence in tumor tissues.

Neither the IFN/ISG median-split Kaplan–Meier analysis nor the multivariable Cox models (unadjusted, clinically adjusted, or adjusted with ESTIMATE microenvironment scores) yielded statistically significant associations with overall survival or progression-free interval after Benjamini–Hochberg correction (Figure 6 and Table 6). This negative finding is informative rather than merely null, as it points to specific limitations in signature design and data context rather than the absence of a biologically relevant association. In detail, the signatures used herein were short (9–15 genes) and derived from a single in vitro infection model, which cannot capture the full complexity of these transcriptional programs in heterogeneous tumor tissues. TCGA-OV expression profiles are bulk measurements influenced by not only the tumor cells, but also the variable immune and stromal infiltration, importantly, ESTIMATE-based deconvolution confirmed that the ECM signature score is strongly driven by stromal cell abundance (StromalScore ρ = 0.83), and inflammatory signatures substantially reflect immune cell infiltration (ImmuneScore ρ = 0.72–0.74 for TNF/NF-κB and NOD/innate), indicating that these scores do not capture infection-specific transcriptional programs independently of microenvironment composition [73]. Furthermore, TCGA-OV is enriched for high-grade serous ovarian carcinoma, a relatively homogeneous and aggressive subtype with limited transcriptomic prognostic stratification in many published signatures, and the analytic cohort may be underpowered to detect modest effect sizes [74,75,76]. These findings do not exclude the possibility that refined or expanded signatures, validated in independent cohorts, could reveal meaningful prognostic associations. The TCGA component should therefore be interpreted as a supportive translational context for pathway-level plausibility rather than as evidence of CT-driven prognostic stratification.

Therefore, several limitations of this study should be acknowledged. This work is entirely in silico and hypothesis-generating, so no experimental validation of candidate pathways or genes has been performed. Experimental confirmation of key findings, for example, using qRT-PCR, Western blotting, or immunostaining in independent infection models or patient-derived samples, would be required to establish biological significance beyond the transcriptomic level. Second, the GSE109428 dataset profiles a single mesenchymal cell population from a limited number of donors (*n* = 3, per condition), which substantially limits statistical power and increases the possibility of donor-specific effects or unstable differential expression estimates. The differential expression results should therefore be treated as hypothesis-generating. Furthermore, the dataset does not include time-matched uninfected controls; both the 24-hpi and 48-hpi comparisons were made against a single set of non-infected controls, and therefore transcriptional changes at each timepoint may partly reflect culture duration effects rather than exclusively infection-specific responses. This limitation is inherent to the original study design and cannot be resolved retrospectively.

Third, the study focuses exclusively on fallopian tube mesenchymal cells, whereas ovarian tumorigenesis involves complex interactions among epithelial, stromal, and immune compartments. The transcriptional programs identified here are cell-type-specific and may not be representative of responses in fallopian tube epithelial cells, which are considered the predominant cell of origin for high-grade serous ovarian carcinoma. The choice of mesenchymal cells is justified by their established roles in stromal remodeling, fibrosis, and inflammatory crosstalk, but the translational relevance of these findings to epithelial-driven carcinogenesis remains to be determined.

Also, functional enrichment and network analyses were performed using g:Profiler and STRING, which rely on curated annotation databases and association evidence that may not fully capture context-specific or cell-type-specific biology, and are subject to annotation biases toward extensively studied genes and pathways. Complementary unbiased approaches, such as pathway topology analysis or network centrality metrics, could provide additional interpretive value in future analyses.

Moreover, the four ssGSEA signatures used for TCGA-OV scoring were short (9–15 genes each) and derived from a single in vitro infection dataset, which limits their generalizability. Although signature genes were selected through a data-driven approach combining g:Profiler enrichment and STRING network evidence, the signatures were not benchmarked against existing immune or ECM signatures in cancer, nor validated across multiple infection datasets. ESTIMATE-based deconvolution confirmed that the ECM signature score is strongly correlated with stromal cell abundance (StromalScore ρ = 0.83), and inflammatory signatures substantially reflect immune cell infiltration, indicating that these scores may not capture infection-specific transcriptional programs independently of microenvironment composition.

Finally, the TCGA-OV survival analyses were exploratory. Although clinically adjusted Cox models incorporating age, tumor stage, histological grade, and residual disease were fitted, and *p*-values were corrected using the Benjamini–Hochberg procedure, no significant associations were identified. The analytic cohort was restricted to samples with complete clinical covariate data (*n* = 264), and results should be interpreted with caution, given the limited signature size, cohort heterogeneity, and absence of CT exposure data in TCGA clinical annotations. The TCGA analyses cannot establish whether transcriptional programs observed in tumors are related to prior CT infection, as pathogen exposure history is unavailable.

In line with these limitations, we propose that future studies should prioritize experimental validation of candidate pathways using more complex and cell-type-specific models, such as co-culture systems incorporating tumor, stromal, and immune cells, to better recapitulate the complex in vivo biology of the tumor microenvironment and its dynamics. Future work combining serological exposure data with tumor transcriptomic profiling in well-annotated cohorts could help bridge the gap between population-level associations and molecular mechanisms. Additionally, expanded signature development using multiple infection datasets and validation in independent ovarian cancer cohorts would improve both robustness and potential prognostic utility.

## 5. Conclusions

In summary, this in silico study shows that CT infection in primary human fallopian tube mesenchymal cells is associated with sustained inflammatory and interferon-linked transcriptional programs, including activation of NF-κB/TNF and IL-17 signaling axes, a densely connected ISG module comprising MX1/MX2, IFIT family members, OAS2, ISG15, and IRF7, and coordinated repression of ECM and adhesion networks at 48-hpi. Cytokine hubs centered on IL1A/IL1B, IL6, and CXCL/CCL chemokines represent candidate mediators of the sustained inflammatory state and constitute priority targets for experimental follow-up.

From a translational perspective, analogous inflammatory and innate immune transcriptional states are detectable in TCGA ovarian tumors, consistent with the biological plausibility of infection-linked programs as features of the tumor microenvironment, though this co-occurrence does not imply a causal relationship with CT infection. Although the short gene signatures derived here did not yield robust prognostic signals in TCGA-OV, a finding that remained consistent across unadjusted, clinically adjusted, and microenvironment-adjusted models, this reflects the exploratory nature of the analysis rather than the absence of a biologically relevant relationship, and does not preclude meaningful associations in larger, better-annotated cohorts with expanded signatures.

Altogether, this work provides a reproducible integrative framework bridging in vitro infection biology and tumor transcriptomics, and identifies specific candidate programs, including cytokine hubs centered on IL1A/IL1B and IL6, the ISG module comprising MX1, IFIT family members, and ISG15, and the ECM repression program centered on collagen and laminin genes. These candidate programs represent tractable entry points for future mechanistic studies in complex cell-type-specific models, including co-culture systems and organoid-based infection models. From an epidemiological perspective, future studies combining serological CT exposure data with tumor transcriptomic profiling in prospectively annotated cohorts with documented infection history would help bridge the gap between population-level associations and molecular mechanisms, and more directly address the potential role of CT infection as a co-factor in female reproductive tract tumorigenesis.

## Figures and Tables

**Table 1:** Summary of experimental design and metadata of the GSE109428 dataset [38].

	Experimental Design and Available Metadata
GEO accession dataset	GSE109428
Platform	GPL21272, Agilent-048908 8×60K whole genome incl V1-V2 linc BioVacSafe final 048908
Species	*Homo sapiens*
Cell type	Primary mesenchymal cells from the human fallopian tube
Infection conditions	*Chlamydia trachomatis* serovar L2 infection vs. uninfected
Time points	24- and 48-hpi
n (control)	3
n (24-hpi)	3
n (48-hpi)	3
Donor information	Primary cells derived from surgical specimens of patients with benign gynecological disease
GEO samples accessions	GSM2942799–GSM2942807

GEO: Gene Expression Omnibus; hpi: hours post-infection.

**Table 2:** Summary of differentially expressed gene sets (FDR < 0.05; |log2FC| ≥ 1).

Gene Set	Upregulated	Downregulated
24-hpi vs. uninfected	238	10
48-hpi vs. uninfected	1112	463
Sustained	223	6
48-hpi-only (late response)	889	457

hpi: hours post-infection.

**Table 3:** Summary of functional enrichment analysis of sustained upregulated genes by CT infection using g:Profiler.

Ontology/Source	Term Name	Term ID	adjP	Term Size	Intersection Size	Genes
GO:BP	Negative regulation of the MAPK cascade	GO:0043409	8.58 × 10^−6^	152	13	*ATF3*, *LIF*, *SPRY2*, *DUSP8*, *PTPRR*, *PSCA*, *DUSP5*, *FLCN*, *DUSP2*, *IL1B*, *PER1*, *DUSP10*, *BMP2*
GO:BP	Cellular response to IL1	GO:0071347	6.09 × 10^−4^	81	9	*IL6*, *NR1D1*, *EGR1*, *EDN1*, *NFKBIA*, *CCL2*, *IL1RN*, *CCL5*, *IL1B*
KEGG	TNF signaling pathway	KEGG:04668	1.43 × 10^−11^	112	14	*CREB5*, *FOS*, *PTGS2*, *LIF*, *IL6*, *CXCL2*, *EDN1*, *BIRC3*, *NFKBIA*, *CCL2*, *CSF2*, *CCL5*, *IL1B*, *CXCL1*
KEGG	IL17 signaling pathway	KEGG:04657	8.96 × 10^−9^	91	11	*FOS*, *PTGS2*, *IL6*, *CXCL2*, *FOSB*, *NFKBIA*, *CCL2*, *CSF2*, *CSF3*, *IL1B*, *CXCL1*
KEGG	Cytokine–cytokine receptor interaction	KEGG:04060	3.87 × 10^−8^	278	16	*IL1A*, *LIF*, *IL24*, *IL6*, *CXCL2*, *CCL2*, *CSF2*, *IL1RN*, *CSF3*, *CCL5*, *IL1B*, *IL1RL1*, *CXCL1*, *IL11*, *TNFRSF9*, *BMP2*
KEGG	MAPK signaling pathway	KEGG:04010	6.19 × 10^−6^	297	14	*IL1A*, *PLA2G4C*, *FOS*, *GADD45A*, *RELB*, *DUSP8*, *PTPRR*, *DUSP5*, *GDNF*, *DDIT3*, *AREG*, *DUSP2*, *IL1B*, *DUSP10*
KEGG	NF-kB signaling pathway	KEGG:04064	6.35 × 10^−5^	96	8	*PTGS2*, *GADD45A*, *CXCL2*, *RELB*, *BIRC3*, *NFKBIA*, *IL1B*, *CXCL1*
REAC	IL10 signaling	REAC:R-HSA-6783783	1.93 × 10^−13^	44	12	*IL1A*, *PTGS2*, *LIF*, *IL6*, *CXCL2*, *CCL2*, *CSF2*, *IL1RN*, *CSF3*, *CCL5*, *IL1B*, *CXCL1*
REAC	IFN-α/β signaling	REAC:R-HSA-909733	3.84 × 10^−12^	72	13	*RSAD2*, *OASL*, *EGR1*, *ISG20*, *MX1*, *IFIT2*, *IFIT3*, *IFIT1*, *OAS2*, *IRF7*, *MX2*, *USP18*, *ISG15*
REAC	IL4 and IL13 signaling	REAC:R-HSA-6785807	7.21 × 10^−4^	107	8	*IL1A*, *FOS*, *PTGS2*, *LIF*, *IL6*, *CCL2*, *BCL6*, *IL1B*

GO:BP: Gene Ontology Biological Process; KEGG: Kyoto Encyclopedia of Genes and Genomes; REAC: Reactome; adjP: g:SCS-adjusted *p*-value.

**Table 4:** Summary of functional enrichment analysis of genes downregulated exclusively at 48-hpi (48-hpi-only down), using g:Profiler.

Ontology/Source	Term Name	Term ID	adjP	Term Size	Intersection Size	Genes
GO:BP	ECM organization	GO:0030198	1.12 × 10^−8^	314	31	*COL12A1*, *ITGA8*, *RECK*, *FMOD*, *COL5A1*, *COL5A2*, *COL1A2*, *LAMC1*, *FBLN1*, *COL18A1*, *COL4A2*, *SULF2*, *LAMB1*, *LAMA2*, *LAMB2*, *COL4A1*, *COL16A1*, *ACAN*, *POSTN*, *NID2*, *HMCN1*, *COL15A1*, *COL3A1*, *MMP10*, *CHADL*, *APP*, *FAP*, *NID1*, *CYP1B1*, *PXDN*, *COMP*
KEGG	ECM-receptor interaction	KEGG:04512	2.51 × 10^−7^	89	14	*ITGA8*, *ITGAV*, *COL1A2*, *LAMC1*, *COL4A2*, *ITGA11*, *LAMB1*, *LAMA2*, *LAMB2*, *COL4A1*, *FN1*, *LAMA4*, *COL6A3*, *COMP*
KEGG	Focal adhesion	KEGG:04510	1.98 × 10^−6^	199	19	*ITGA8*, *ITGAV*, *COL1A2*, *LAMC1*, *FLNA*, *COL4A2*, *ITGA11*, *LAMB1*, *LAMA2*, *LAMB2*, *COL4A1*, *FN1*, *VCL*, *LAMA4*, *PRKCA*, *MYLK*, *AKT3*, *COL6A3*, *COMP*
KEGG	Small-cell lung cancer	KEGG:05222	2.97 × 10^−5^	92	12	*ITGAV*, *LAMC1*, *COL4A2*, *LAMB1*, *LAMA2*, *LAMB2*, *COL4A1*, *FN1*, *CCNE2*, *CDKN1B*, *LAMA4*, *AKT3*
REAC	MET activates PTK2 signaling	REAC:R-HSA-8874081	9.22 × 10^−8^	30	10	*COL5A1*, *COL5A2*, *COL1A2*, *LAMC1*, *LAMB1*, *LAMA2*, *LAMB2*, *FN1*, *COL3A1*, *LAMA4*
REAC	Signaling by MET	REAC:R-HSA-6806834	1.53 × 10^−4^	76	11	*COL5A1*, *COL5A2*, *COL1A2*, *LAMC1*, *LAMB1*, *LAMA2*, *LAMB2*, *FN1*, *COL3A1*, *LAMA4*, *EPS15*

GO:BP: Gene Ontology Biological Process; KEGG: Kyoto Encyclopedia of Genes and Genomes; REAC: Reactome; adjP: g:SCS-adjusted *p*-value.

**Table 5:** Summary of functional enrichment analysis of genes upregulated exclusively at 48-hpi (48 hpi-only up), using g:Profiler.

Ontology/Source	Term Name	Term ID	adjP	Term Size	Intersection Size	Genes
KEGG	Transcriptional misregulation in cancer	KEGG:05202	9.27 × 10^−5^	170	21	*DUSP6*, *NFKB1*, *CEBPB*, *REL*, *PLAU*, *MDM2*, *DDB2*, *ITGAM*, *TFE3*, *DOT1L*, *NR4A3*, *AFF1*, *CDKN1A*, *BCL2L1*, *MMP3*, *BCL2A1*, *MITF*, *HMGA2*, *ETV4*, *RARA*, *ZBTB17*
KEGG	Cytokine–cytokine receptor interaction	KEGG:04060	1.05 × 10^−4^	278	28	*IL33*, *CXCR4*, *GDF15*, *INHBA*, *IL23A*, *EDA2R*, *IL13RA2*, *TNFRSF10A*, *TNFRSF10B*, *IL7R*, *FASLG*, *CCL7*, *CCL3*, *IL36B*, *IL32*, *CXCL11*, *RELL1*, *IFNB1*, *IFNE*, *TNFSF14*, *IL6R*, *TNF*, *RELL2*, *CLCF1*, *CXCL3*, *EPOR*, *NGF*, *IL17RE*
REAC	Signaling by interleukins	REAC:R-HSA-449147	2.19 × 10^−4^	452	39	*IL33*, *MMP1*, *DUSP6*, *CASP3*, *NFKB1*, *IL23A*, *SQSTM1*, *PIK3CD*, *RIPK2*, *ICAM1*, *DUSP4*, *TRAF6*, *IL13RA2*, *NFKB2*, *IL7R*, *HGF*, *FASLG*, *ITGAM*, *IL1RAPL1*, *LCP1*, *CCL3*, *IL36B*, *IL32*, *MCL1*, *NFKBIB*, *FOXO3*, *JUNB*, *S1PR1*, *IL6R*, *TNF*, *CDKN1A*, *PELI2*, *BCL2L1*, *MMP3*, *CLCF1*, *STX1A*, *VAMP2*, *ELK1*, *IL17RE*
REAC	IL4 and IL13 signaling	REAC:R-HSA-6785807	6.87 × 10^−4^	107	16	*MMP1*, *IL23A*, *ICAM1*, *IL13RA2*, *HGF*, *FASLG*, *ITGAM*, *MCL1*, *FOXO3*, *JUNB*, *S1PR1*, *IL6R*, *TNF*, *CDKN1A*, *BCL2L1*, *MMP3*

KEGG: Kyoto Encyclopedia of Genes and Genomes; REAC: Reactome; adjP: g:SCS-adjusted *p*-value.

**Table 6:** Multivariable Cox proportional hazards models for OS and PFI in TCGA-OV using ssGSEA signature scores. Three model specifications are shown: unadjusted (signature scores only; *n* = 307); clinically adjusted (additionally including age, tumor stage, histological grade, and residual disease; *n* = 264) and adjusted with ESTIMATE (further incorporating ImmuneScore and StromalScore to account for tumor microenvironment composition; *n* = 264). The reduction from *n* = 307 to *n* = 264 reflects exclusion of samples with missing clinical covariate data, primarily residual disease status. *p*-values were adjusted using the Benjamini–Hochberg false discovery rate procedure. No signature reached statistical significance in any model after correction. Wide confidence intervals for the NOD/innate signature reflect collinearity with other immune signatures and limited statistical power. HR, hazard ratio; CI, 95% confidence interval; FDR, Benjamini–Hochberg adjusted *p*-value.

Signature	Model	OS HR (95% CI)	OS *p*-Value	PFI HR (95% CI)	PFI *p*-Value
ECM	Unadjusted	1.30 (0.22–7.60)	0.767	1.35 (0.28–6.49)	0.706
	Adjusted	0.71 (0.12–4.24)	0.705	1.29 (0.27–6.29)	0.750
	Adj + ESTIMATE	0.09 (0.00–4.12)	0.217	0.38 (0.01–13.99)	0.601
IFN/ISG	Unadjusted	0.69 (0.25–1.87)	0.461	0.80 (0.31–2.10)	0.656
	Adjusted	0.80 (0.27–2.37)	0.689	0.86 (0.31–2.37)	0.774
	Adj + ESTIMATE	0.72 (0.24–2.15)	0.561	0.85 (0.31–2.34)	0.755
TNF/NF-κB	Unadjusted	1.83 (0.39–8.72)	0.446	0.86 (0.19–3.94)	0.844
	Adjusted	1.18 (0.23–6.01)	0.838	0.66 (0.13–3.19)	0.600
	Adj + ESTIMATE	1.16 (0.18–7.73)	0.875	0.82 (0.14–4.95)	0.827
NOD/innate immunity	Unadjusted	2.04 (0.11–39.18)	0.637	4.77 (0.27–84.27)	0.286
	Adjusted	2.21 (0.09–52.21)	0.623	6.49 (0.33–129.25)	0.220
	Adj + ESTIMATE	4.37 (0.09–206.90)	0.453	16.36 (0.40–675.03)	0.141

Unadjusted models: *n* = 307 (OS events = 185; PFI events = 214). Clinically adjusted and Adj + ESTIMATE models: *n* = 264 (OS events = 160; PFI events = 186); 43 samples excluded due to missing clinical covariate data, primarily residual disease status. All *p*-values are unadjusted, Benjamini–Hochberg correction was applied across all models and endpoints; no signature reached significance after correction. Wide confidence intervals for NOD/innate immunity reflect collinearity and limited statistical power.

## Data Availability

The original contributions presented in this study are included in the article/Supplementary Material. Further inquiries can be directed to the corresponding author.

## Supplementary Materials

The following supporting information can be downloaded at: https://www.mdpi.com/article/10.3390/cancers18121920/s1. Supplementary File S1: R script containing the pipeline used for the analysis of the dataset GSE109428; Supplementary File S2: R script containing the pipeline used for the TCGA-OV analysis.

## Abbreviations

The following abbreviations are used in this manuscript:

CT	*Chlamydia trachomatis*
AdjP	Adjusted *p*-values
Log2FC	Log_2_ fold change
GO:BP	Gene Ontology Biological Process
PPI	Protein–protein interaction
STRING	Search Tool for the Retrieval of Interacting Genes/Proteins
NCBI	National Center for Biotechnology Information
DEG	Differentially Expressed Genes
p.i.	Post-infection
GEO	Gene Expression Omnibus
PID	Pelvic inflammatory disease
